# Tolerance Modelling of Vibrations of a Sandwich Plate with Honeycomb Core

**DOI:** 10.3390/ma15217611

**Published:** 2022-10-29

**Authors:** Jakub Marczak

**Affiliations:** Department of Structural Mechanics, Łódź University of Technology, 90-924 Łódź, Poland; jakub.marczak@p.lodz.pl

**Keywords:** sandwich plate, vibrations, honeycomb core, tolerance modelling, periodic microstructure

## Abstract

Sandwich structures are commonly used in many branches of modern engineering, such as aerospace or naval constructions. In this work, a vibration analysis of such structures is performed with the use of an anlytical model based on a zig-zag hypothesis. Due to the assumed periodic microstructure, which may occure in any layer of the structure, the initial governing equations describing its dynamic behaviour may contain periodic, non-continuous coefficients. The main aim of the presented paper is to show an analytical solution to the issue of the vibration analysis of the mentioned structures. With the use of the tolerance averaging technique, the initial governing equations are transformed to the form with constant coefficients, which is convenient to solve using well-known mathematical methods. The derived model is a versatile solution for any type of periodically inhomogeneous sandwich plate, including sandwich plates with a honeycomb core. Eventually, in the calculation example, the application of the derived averaged model in the analysis of vibrations of such structures is presented and discussed. The convergence of results of the tolerance model and FEM analysis proves the correctness and superiority of the proposed solution.

## 1. Introduction

Sandwich structures are a specific group of composites, which usually consist of at least three layers. Two outer layers, so called *faces*, are characterised by high mechanical properties, while an inner layer, the so called *core*, can be specially formed to produce a higher stiffness of the whole structure, desired thermal or acoustic insulation or heat resistance. Due to their exceptional strength-to-weight ratio, they are commonly used in aerospace, naval or even civil engineering.

Even though their properties have been known for decades, there are still many groups of researchers who investigate such structures. Let us mention several interesting recent papers, such as static analysis of sandwich structure with corrugated core Tewari et al. [1], multi-scale static analysis of sandwich composites [2], the analysis of dynamic response of sandwich structures to various impacts [3,4,5,6,7] or the attempt of creating the easy-to-repair core [8]. Among those papers, one can find a significant number of works, which are focused on an experimental examination of certain properties of the composite. The importance of such works is unquestionable. However, the preparation of a specific experiment is always a time-consuming and costly process, which does not necessarily have to end up with reliable results. This is why the ability to create computational models of the considered structures is vitally important.

There are many methods of modelling sandwich structures. Among the analytical approaches one can distinguish classic plate theory, broken line hypothesis (zigzag theory), refined zigzag theory, sinusoidal shear deformation theory, first-order shear deformation theory or higher-order shear deformation theory. The differences between each of those theories are widely described in the literature, cf. [9,10,11,12], while their applications can be found, for example, in [13,14,15]. The mentioned analytical models of sandwich structures usually produce precise results only if the layers of the considered structure are homogeneous. Meanwhile, the cores of the sandwich structures are often designed as laminates, composites or even corrugated and lattice-type structures. In such cases, the most common and versatile method of analysis is the finite element method. The application of this method can be found, for example, in [16,17,18,19,20,21]. However, it should be mentioned that creating and evaluating numerical models of sandwich plates with complicated heterogeneous cores is a time-consuming process, which additionally requires a lot of computing resources.

**Figure 1:**
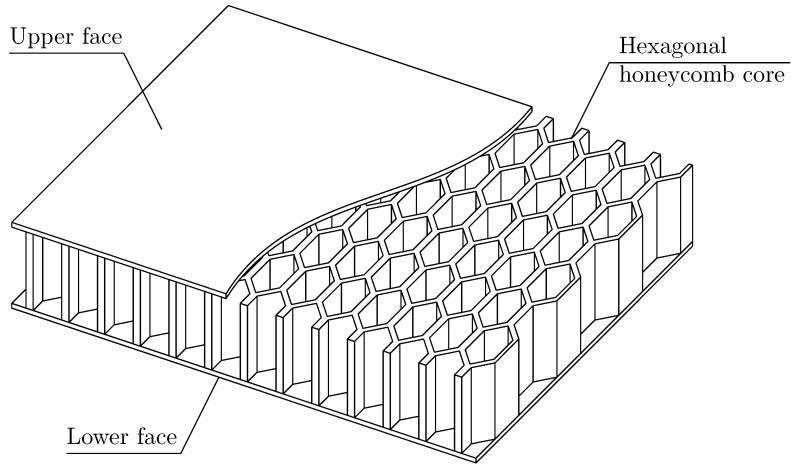
Sandwich plate with hexagonal honeycomb core.

In this paper, an issue of dynamics of a specific type of sandwich plate with a hexagonal honeycomb core is investigated, cf. Figure 1. Due to the complicated form of the core, the most common approach to modelling such structures is the evaluation of effective properties of the inner layer, cf. [22,23,24]. Such an approach can be considered an effective method for obtaining the general overall performance of the considered structure. However, it neglects local fluctuations of stress and strains, which occur within the faces due to the heterogeneous core. In this paper, the analytical model, which takes into consideration the mentioned fluctuations, is presented and validated. Basing the broken line hypothesis on displacements, the initial governing equations of the three-layered sandwich structure are derived. Due to the heterogeneous core, their coefficients are a periodic, non-continuous and highly-oscillating function of the spatial coordinates. In order to overcome the difficulties in solving such system of partial differential equations, the tolerance averaging technique is used. With its concepts, it is possible to transform the initial system of partial differential equations with periodic coefficients into the form with constant coefficients, cf. [25]. Within the literature, one can find multiple applications of this technique in various mechanical issues, such as stability analysis [26,27,28,29], dynamics [30,31,32,33] or even heat conduction issues [34,35,36,37].

## 2. Modelling Foundations

**Figure 2:**
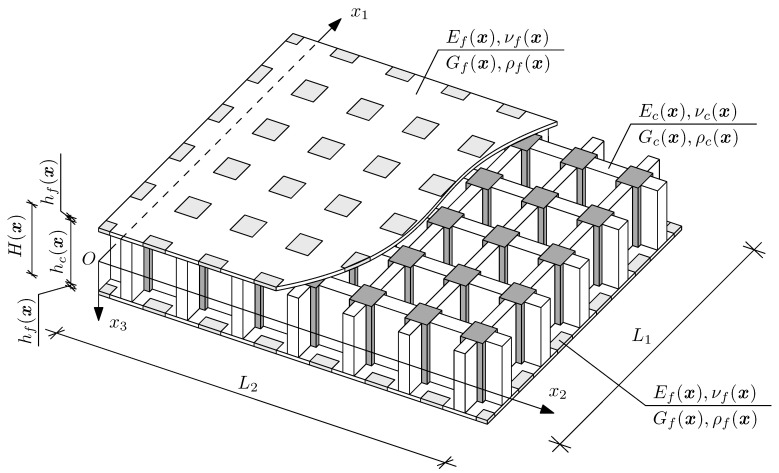
Sandwich plate with periodic microstructure.

Let us denote $Ox_{1}x_{2}x_{3}$ as an orthogonal Cartesian coordinate system, where $x\equiv \left(x_{1},x_{2}\right)$, and *t* as a time coordinate. The considered three-layered plate is assumed to be rectangular and to have spans $L_{1}$ and $L_{2}$, along $x_{1}$- and $x_{2}$-axis directions, respectively. As a result, the mid-plane of the core of the structure can be denoted $\Pi =\left[0,L_{1}\right]\times \left[0,L_{2}\right]$. Moreover, the considered structure is assumed to be symmetric toward the mentioned mid-plane; hence, for the specific x coordinate, both outer layers have the same material properties and thicknesses. Eventually, let us introduce $h_{c}\left(x\right)$ as the thickness of the core, $h_{f}\left(x\right)$ as a thickness of the outer layers and $H\left(x\right)=h_{c}\left(x\right)+2h_{f}\left(x\right)$ as a total thickness of the sandwich plate, so it is possible to denote the whole region occupied by the undeformed structure as: $\Omega \equiv \left\{\left(x,x_{3}\right):-H\left(x\right)/2\leq x_{3}\leq H\left(x\right)/2,x\in \Pi \right\}$, cf. Figure 2.

At this stage of investigations, let us assume that every layer of the sandwich plate can be characterised by a specific periodic microstructure. It means that all material properties and thicknesses of every layer can be a periodic function of spatial coordinate $x\equiv \left(x_{1},x_{2}\right)$. Based on this assumption, it is possible to distinguish a small repeatable element called *periodicity cell* Δ. The periodicity cell can be given by any shape. In the easiest 2D case, it is defined as a rectangle, in which the dimensions along the $x_{1}$- and $x_{2}$-axis directions are defined as $l_{1}$ and $l_{2}$, respectively.

Eventually, for the sake of simplicity, let us assume that the whole structure is made of isotropic materials. Consequently, let us denote $E_{f}\left(x\right),\nu_{f}\left(x\right),G_{f}\left(x\right),\rho_{f}\left(x\right)$ as modulus of elasticity, Poisson ratio, shear modulus and mass density of the faces, and $E_{c}\left(x\right),\nu_{c}\left(x\right),G_{c}\left(x\right),\rho_{c}\left(x\right)$ as modulus of elasticity, Poisson ratio, shear modulus and mass density of the core.

## 3. Derivation of Initial Governing Equations

In this section, the initial governing equations describing the dynamic behaviour of the already described structure are derived and discussed. In all subsequent equations, the spatial derivative is denoted $\partial_{i}\equiv \frac{\partial }{\partial x_{i}},i=1,2,3$, while a time derivative is denoted with an overdot.

**Figure 3:**
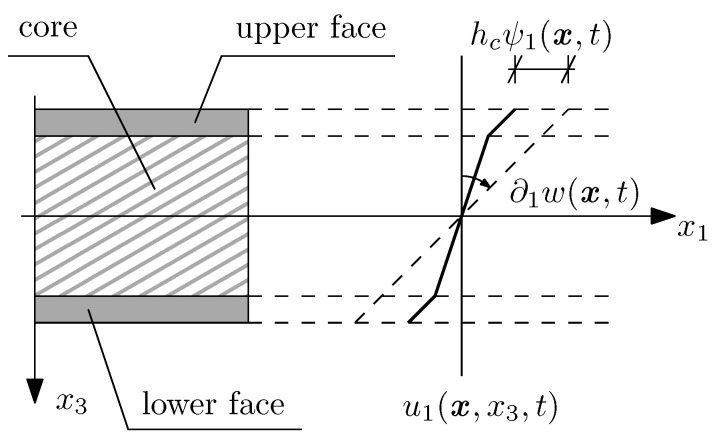
In-plane displacement hypothesis according to the broken line hypothesis.

Let us start with the formulation of the in-plane deformation hypothesis for the considered plate. In this paper, the broken line hypothesis (or the zigzag hypothesis) is applied to the analysis of vibrations. According to this, the displacements along each spatial coordinate $\left(x_{1},x_{2},x_{3}\right)$ are defined with specific linear piecewise functions as follows:

(1) $$\begin{array}{cc} u_{1}\left(x,x_{3},t\right) & =\left\{\begin{array}{cc} -x_{3}\partial_{1}w\left(x,t\right)-h_{c}\psi_{1}\left(x,t\right) & -\frac{H}{2}\leq x_{3}<-\frac{h_{c}}{2}\\ -x_{3}\partial_{1}w\left(x,t\right)+2x_{3}\psi_{1}\left(x,t\right) & -\frac{h_{c}}{2}\leq x_{3}\leq \frac{h_{c}}{2}\\ -x_{3}\partial_{1}w\left(x,t\right)+h_{c}\psi_{1}\left(x,t\right) & \frac{h_{c}}{2}<x_{3}\leq \frac{H}{2} \end{array}\right.,\\ u_{2}\left(x,x_{3},t\right) & =\left\{\begin{array}{cc} -x_{3}\partial_{2}w\left(x,t\right)-h_{c}\psi_{2}\left(x,t\right) & -\frac{H}{2}\leq x_{3}<-\frac{h_{c}}{2}\\ -x_{3}\partial_{2}w\left(x,t\right)+2x_{3}\psi_{2}\left(x,t\right) & -\frac{h_{c}}{2}\leq x_{3}\leq \frac{h_{c}}{2}\\ -x_{3}\partial_{2}w\left(x,t\right)+h_{c}\psi_{2}\left(x,t\right) & \frac{h_{c}}{2}<x_{3}\leq \frac{H}{2} \end{array}\right.,\\ u_{3}\left(x,x_{3},t\right) & \equiv w(x,t), \end{array}$$

where w(x,t) is a function of vertical displacements of the mid-plane of the structure Π, while $\psi_{1}\left(x,t\right),\psi_{2}\left(x,t\right)$ are certain dimensionless functions representing variations of in-plane displacements caused by three-layered structure. The physical sense of those functions is shown in Figure 3.

The deformation hypothesis should be followed by a small deformation assumption:

$$\varepsilon_{ij}=\frac{1}{2}\left(\partial_{j}u_{i}+\partial_{i}u_{j}\right),\;i,j=1,2,3,$$

and a stress-strain relation. In our case, a typical Hooke’s law for plates made of isotropic materials is used, so:

$$\sigma_{\alpha \beta }={\tilde{C}}_{\alpha \beta \gamma \delta }\varepsilon_{\gamma \delta },\;\alpha ,\beta ,\gamma ,\delta =1,2,$$

where the only non-zero terms are:

$$\begin{array}{cc} {\tilde{C}}_{1111} & ={\tilde{C}}_{2222}=\frac{E(x,x_{3})}{1-{\left[\nu (x,x_{3})\right]}^{2}},\\ {\tilde{C}}_{1122} & ={\tilde{C}}_{2211}=\frac{E\left(x,x_{3}\right)\cdot \nu \left(x,x_{3}\right)}{1-{\left[\nu (x,x_{3})\right]}^{2}},\\ {\tilde{C}}_{1212} & ={\tilde{C}}_{2121}={\tilde{C}}_{1221}={\tilde{C}}_{2112}=G\left(x,x_{3}\right)=\frac{E(x,x_{3})}{2\left[1+\nu (x,x_{3})\right]}, \end{array}$$

and:

$$E\left(x,x_{3}\right)=\left\{\begin{array}{c} E_{f}\left(x\right)\;for\;-H/2\leq x_{3}<-h_{c}/2\\ E_{c}\left(x\right)\;for\;-h_{c}/2\leq x_{3}\leq h_{c}/2\\ E_{f}\left(x\right)\;for\;h_{c}/2<x_{3}\leq H/2 \end{array},\right.$$

$$\nu \left(x,x_{3}\right)=\left\{\begin{array}{c} \nu_{f}\left(x\right)\;for\;-H/2\leq x_{3}<-h_{c}/2\\ \nu_{c}\left(x\right)\;for\;-h_{c}/2\leq x_{3}\leq h_{c}/2\\ \nu_{f}\left(x\right)\;for\;h_{c}/2<x_{3}\leq H/2 \end{array}.\right.$$

Eventually, one should formulate the equations of equilibrium, which, for the small part of the considered plate, can be written as:

$$\begin{array}{cc} \partial_{\alpha \beta }M_{\alpha \beta }+q & =\mu {\ddot{u}}_{3}+\partial_{\alpha }{\ddot{\tilde{\mu }}}_{\alpha },\\ \partial_{\beta }M_{\alpha \beta }-Q_{\alpha }+m_{\alpha } & ={\ddot{\tilde{\mu }}}_{\alpha },\\ \alpha ,\beta & =1,2, \end{array}$$

where:

$$\begin{array}{cc} M_{\alpha \beta }\left(x,t\right)=\int_{H(x)}\sigma_{\alpha \beta }\left(x,x_{3},t\right)x_{3}dx_{3}, & Q_{\alpha }\left(x,t\right)=\int_{H(x)}\sigma_{\alpha 3}\left(x,x_{3},t\right)dx_{3},\\ \mu \left(x\right)=\int_{H(x)}\rho \left(x,x_{3}\right)dx_{3}, & {\tilde{\mu }}_{\alpha }\left(x,t\right)=\int_{H(x)}\rho \left(x,x_{3}\right){\ddot{u}}_{\alpha }\left(x,x_{3},t\right)x_{3}dx_{3},\\ q\left(x,t\right)=\sigma_{33}\left(x,x_{3},t\right){|}_{-H(x)/2}^{H(x)/2}+\partial_{\alpha }m_{\alpha }\left(x,t\right), & m_{\alpha }\left(x,t\right)=\sigma_{\alpha 3}\left(x,x_{3},t\right)x_{3}{|}_{-H(x)/2}^{H(x)/2}. \end{array}$$

As a result of all the presented relations, it is possible to derive a set of initial governing equations of the three-layered sandwich plate, which can be presented in a simplified form as:

(2) $$\begin{array}{cc} \partial_{\alpha \beta }\left[C_{\alpha \beta \gamma \delta }\partial_{\gamma \delta }w\left(x,t\right)-{\hat{C}}_{\alpha \beta \gamma \delta }\partial_{\gamma }\psi_{\delta }\left(x,t\right)\right]-A_{11}\left(\rho_{f},\rho_{c}\right)\partial_{\alpha \alpha }\ddot{w}\left(x,t\right)\\ +A_{12}\left(\rho_{f},\rho_{c}\right)\partial_{\alpha }{\ddot{\psi }}_{\alpha }\left(x,t\right)+B_{1}\ddot{w}\left(x,t\right) & =q\left(x,t\right)/h_{c}^{3},\\ \partial_{\alpha \gamma }\left[C_{\alpha \beta \gamma \delta }\partial_{\beta }w\left(x,t\right)-{\hat{C}}_{\alpha \beta \gamma \delta }\psi_{\beta }\left(x,t\right)\right]-A_{11}\left(\rho_{f},\rho_{c}\right)\partial_{\delta }\ddot{w}\left(x,t\right)\\ +A_{12}\left(\rho_{f},\rho_{c}\right){\ddot{\psi }}_{\delta }\left(x,t\right)+B_{2}\psi_{\delta }\left(x,t\right) & =m_{\delta }\left(x,t\right)/h_{c}^{3},\\ \alpha ,\beta ,\gamma ,\delta & =1,2, \end{array}$$

where:

(3) $$\begin{array}{cc} a_{1}=\left(\frac{2}{3}X^{2}+X+\frac{1}{2}\right)X, & a_{2}=X^{2}+X,\;\;\;\;X=\frac{h_{f}\left(x\right)}{h_{c}\left(x\right)},\\ A_{11}\left(Y,Z\right)=Y\cdot a_{1}+\frac{1}{12}\cdot Z, & A_{12}\left(Y,Z\right)=Y\cdot a_{2}+\frac{1}{6}\cdot Z,\\ B_{1}=\frac{2\cdot \rho_{f}\left(x\right)\cdot h_{f}\left(x\right)+\rho_{c}\left(x\right)\cdot h_{c}\left(x\right)}{{\left[h_{c}\left(x\right)\right]}^{3}}, & B_{2}=\frac{2\cdot G_{c}\left(x\right)}{{\left[h_{c}\left(x\right)\right]}^{2}},\\ C_{1111}=C_{2222}=A_{11}\left(\frac{E_{f}\left(x\right)}{1-{\left[\nu_{f}\left(x\right)\right]}^{2}},\frac{E_{c}\left(x\right)}{1-{\left[\nu_{c}\left(x\right)\right]}^{2}}\right), & {\hat{C}}_{1111}={\hat{C}}_{2222}=A_{12}\left(\frac{E_{f}\left(x\right)}{1-{\left[\nu_{f}\left(x\right)\right]}^{2}},\frac{E_{c}\left(x\right)}{1-{\left[\nu_{c}\left(x\right)\right]}^{2}}\right),\\ C_{1212}=C_{1221}=C_{2121}=C_{2112}=A_{11}\left(G_{f}\left(x\right),G_{c}\left(x\right)\right), & {\hat{C}}_{1212}={\hat{C}}_{1221}={\hat{C}}_{2121}={\hat{C}}_{2112}=A_{12}\left(G_{f}\left(x\right),G_{c}\left(x\right)\right),\\ C_{1122}=C_{2211}=A_{11}\left(\frac{E_{f}\left(x\right)\nu_{f}\left(x\right)}{1-{\left[\nu_{f}\left(x\right)\right]}^{2}},\frac{E_{c}\left(x\right)\nu_{c}\left(x\right)}{1-{\left[\nu_{c}\left(x\right)\right]}^{2}}\right), & {\hat{C}}_{1122}={\hat{C}}_{2211}=A_{12}\left(\frac{E_{f}\left(x\right)\nu_{f}\left(x\right)}{1-{\left[\nu_{f}\left(x\right)\right]}^{2}},\frac{E_{c}\left(x\right)\nu_{c}\left(x\right)}{1-{\left[\nu_{c}\left(x\right)\right]}^{2}}\right). \end{array}$$

Equations (2), together with the subsequent denotations (3), constitute the initial analytical model of dynamic behaviour of the three-layered sandwich plate based on the broken line hypothesis. Based on the definitions (3) it can be observed that in the case of structure with a certain type of periodic inhomogeneity, the system of Equations (2) is characterised by periodic, non-continuous and highly oscillating coefficients, which makes it very difficult to solve. In the next step of investigations, the tolerance averaging technique is applied to transform the obtained system of equations into the form with constant coefficients.

## 4. Basics of Tolerance Averaging Technique

In this section, only the main concepts of the tolerance averaging technique are presented. For the detailed description of the used technique, one should reference the literature, for example, [25].

Let us start with a definition of *a tolerance parameter* δ, which is an arbitrary positive number. In the whole modelling process it is assumed that certain terms with a difference smaller than the tolerance parameter δ can be treated as equals.

Let us distinguish two points from the mid-plane of the considered structure as $x=\left(x_{1},x_{2}\right)$ and $x^{\prime }=\left(x_{1}^{\prime },x_{2}^{\prime }\right)$. The specific periodicity cell Δ with a centre at x is denoted as $\Delta \left(x\right)=x+\Delta$, where, in the case of a rectangular periodicity cell $\Delta =\left[-l_{1}/2,l_{1}/2\right]\times \left[-l_{2}/2,l_{2}/2\right]$. The close surroundings of such a cell are defined as $\Pi_{x}=\Pi \cap \underset{x^{\prime }\in \Delta \left(x\right)}{⋃}\Delta \left(x^{\prime }\right),x\in \bar{\Pi }$, where $\bar{\Pi }=\underset{x^{\prime }\in \Delta \left(x\right)}{⋃}\Delta \left(x^{\prime }\right),x\in \Pi$. Keeping in mind those denotations, it is possible to define different types of functions, such as *tolerance periodic function*, *slowly varying function*, *highly oscillating function* and *fluctuation shape function*, which are crucial for the tolerance averaging technique.

Let $H^{2}\left(\Pi \right)$ be a Sobolev space, $\partial^{k}f$ be the *k*^th^ gradient of function $f=f\left(x\right),x\in \Pi ,k=0,1,2,\ldots ,n$, where $\partial^{0}f=f$.

Function $f\in H^{2}\left(\Pi \right)$ is called *the tolerance periodic function* with respect to cell Δ and tolerance parameter δ, $f\in TP_{\delta }^{k}\left(\Delta \right)$, if the following conditions are held:

- $\left(\forall x\in \Pi \right)\left[\exists {\tilde{f}}^{\left(k\right)}\left(x,\cdot \right)\in H^{0}\left(\Delta \right)\right]\left[{∥{\left.\partial^{k}f\right|}_{\Pi_{x}}-{\tilde{f}}^{\left(k\right)}\left(x,\cdot \right)∥}_{H^{0}\left(\Pi_{x}\right)}\leq \delta \right]$,
- $\int_{\Delta \left(\cdot \right)}{\tilde{f}}^{\left(k\right)}\left(\cdot ,z\right)dz\in C^{0}\left(\bar{\Pi }\right).$

Function ${\tilde{f}}^{\left(k\right)}\left(x,\cdot \right)$ is referred to as *the periodic approximation* of $\partial^{k}f$ in $\Delta \left(x\right),x\in \Pi$.

Function $v\in H^{2}\left(\Pi \right)$ is called *the slowly varying function* with respect to cell Δ and tolerance parameter δ, $v\in SV_{\delta }^{k}\left(\Delta \right)$, if the following conditions are held:

- $v\in TP_{\delta }^{k}\left(\Delta \right)$,
- $\left(\forall x\in \Pi \right)\left[{\left.{\tilde{v}}^{\left(k\right)}\left(x,\cdot \right)\right|}_{\Delta \left(x\right)}=\partial^{k}v\left(x,\cdot \right)\right].$

Function $h\in H^{2}\left(\Pi \right)$ is called *the highly oscillating function* with respect to cell Δ and tolerance parameter δ, $h\in HO_{\delta }^{k}\left(\Delta \right)$, if the following conditions are held:

- $h\in TP_{\delta }^{k}\left(\Delta \right)$,
- $\left(\forall x\in \Pi \right)\left[{\left.{\tilde{h}}^{\left(k\right)}\left(x,\cdot \right)\right|}_{\Delta \left(x\right)}=\partial^{k}\tilde{h}\left(x,\cdot \right)\right].$

Function $g\in H^{2}\left(\Pi \right)$ is called *the fluctuation shape function* with respect to cell Δ and tolerance parameter δ, $g\in FS_{\delta }^{k}\left(\Delta \right)$, if it depends on *the microstructure parameter l* and:

- $g\in HO_{\delta }^{k}\left(\Delta \right)$,
- $\partial^{k}g\in O\left(l^{n-k}\right),$
- $\forall \left(x\in \Pi :\Delta \left(x\right)\subset \Pi \right)\left[\langle g\Gamma \rangle \left(x\right)=0\right],$

where Γ is a certain periodic and positive function.

There are several original concepts used in the tolerance modelling procedure. One of them is the concept of *an averaging operator*, which for a 2D issue can be presented in the form:

(4) $$\langle \partial^{k}f\rangle \left(x\right)=\frac{1}{\left|\Delta \right|}\int_{\Delta \left(x\right)}{\tilde{f}}^{\left(k\right)}\left(x,y\right)dy,\;k=0,1,2,\ldots ,n,\;x\in \bar{\Pi }.$$

Another concept is called *a micro–macro decomposition* of a certain physical field. According to its certain field, u(·,t) can be expressed as a sum of an averaged macro-field U(·,t) of a certain physical property, $U\left(\cdot ,t\right)\in SV_{\delta }^{k}\left(\Delta \right)$, and products of arbitrarily chosen fluctuation shape functions $h^{A}\left(\cdot \right)$, $h^{A}\left(\cdot \right)\in FS_{\delta }^{k}\left(\Delta \right)$, and unknown functions of fluctuation amplitudes $V^{A}\left(\cdot ,t\right)$, $V^{A}\left(\cdot ,t\right)\in SV_{\delta }^{k}\left(\Delta \right)$:

(5) $$u\left(\cdot ,t\right)=U\left(\cdot ,t\right)+h^{A}\left(\cdot \right)V^{A}\left(\cdot ,t\right),\;A=1,2,\ldots ,n.$$

Eventually, based on all of the aforementioned concepts, a set of tolerance averaging approximations can be formulated. Exemplary transformations are presented below:

(6) $$\begin{array}{cc} \langle \phi \rangle \left(\cdot \right) & =\langle \tilde{\phi }\rangle \left(\cdot \right)+O\left(\delta \right),\\ \langle \phi F\rangle \left(\cdot \right) & =\langle \phi \rangle \left(\cdot \right)F\left(\cdot \right)+O\left(\delta \right),\\ \langle \phi \partial^{k}\left(gF\right)\rangle \left(\cdot \right) & =\langle \phi \partial^{k}g\rangle \left(\cdot \right)F\left(\cdot \right)+O\left(\delta \right),\\ \langle g\partial^{k}\left(\phi \Phi \right)\rangle \left(\cdot \right) & =-\langle \phi \Phi \partial^{k}g\rangle \left(\cdot \right)+O\left(\delta \right),\\ k=1,2,\ldots ,n, & \;0<\delta \ll 1,\\ \phi ,\Phi \in TP_{\delta }^{k}\left(\Delta \right), & \;F\in SV_{\delta }^{k}\left(\Delta \right),\;g\in FS_{\delta }^{k}\left(\Delta \right), \end{array}$$

where $O\left(\delta \right)$ is a negligibly small term.

## 5. Governing Equations of the Tolerance Model

In this section, a general procedure for deriving a tolerance model of vibrations of a micro-heterogeneous three-layered sandwich plate is presented and discussed.

Let us start with the initial system of governing Equations (2). As was already mentioned, in the case of a structure with specific periodic inhomogeneities, the system of Equations (2) is characterised by periodic, non-continuous coefficients. The presented tolerance modelling procedure makes it possible to transform it to a system of differential equations with constant coefficients.

At the beginning of the transformations, the whole considered structure is divided into a number of small, repeatable elements, called periodicity cells Δ. The system of governing equations remains true for any basic periodicity cell. In the next step, the micro-macro decomposition of all displacement fields present in (2) is required. Basing on definition (5), let us formulate it as follows:

(7) $$\begin{array}{cc} w(x,t) & =W\left(x,t\right)+g^{A}\left(x\right)Q^{A}\left(x,t\right),\\ \psi_{\alpha }\left(x,t\right) & =\Theta_{\alpha }\left(x,t\right)+h_{\alpha }^{B}\left(x\right)\Phi_{\alpha }^{B}\left(x,t\right),\\ \alpha & =1,2,\;A=1,2,\ldots ,N,\;B=1,2,\ldots ,M, \end{array}$$

where $W\left(x,t\right)\in SV_{\delta }^{4},$ $\Theta_{\alpha }\left(x,t\right)\in SV_{\delta }^{3}$ are vertical and in-plane macrodisplacements, respectively, $g^{A}\left(x\right)\in FS_{\delta }^{4}\left(\Delta \right),$ $h_{\alpha }^{B}\left(x\right)\in FS_{\delta }^{3}\left(\Delta \right)$ are vertical and in-plane fluctuation shape functions and $Q^{A}\left(x,t\right)\in SV_{\delta }^{4},$ $\Phi_{\alpha }^{B}\left(x,t\right)\in SV_{\delta }^{3}$ are amplitudes of those fluctuations. Then, the averaging operator (4) should be applied to the derived system of equations and the orthogonalisation condition of those equations and an arbitrarily chosen set of fluctuation shape functions should be formulated. Eventually, a series of tolerance averaging approximations (6) should be applied, so the most convenient form of governing equations is obtained.

As a result of all the aforementioned transformations, the tolerance model of vibrations of three-layered periodic sandwich plate can be written in the following form:

(8) $$\begin{array}{cc} \langle C_{\alpha \beta \gamma \delta }\rangle \partial_{\alpha \beta \gamma \delta }W+\langle C_{\alpha \beta \gamma \delta }\partial_{\alpha \beta \gamma \delta }g^{A}\rangle Q^{A}-\langle {\hat{C}}_{\alpha \beta \gamma \delta }\rangle \partial_{\alpha \beta \gamma }\Theta_{\delta }-\langle {\hat{C}}_{\alpha \beta \gamma \delta }\partial_{\alpha \beta \gamma }h_{\delta }^{B}\left(x\right)\rangle \Phi_{\delta }^{B}+\\ -\langle A_{11}\left(\rho_{f},\rho_{c}\right)\rangle \partial_{\alpha \alpha }\ddot{W}-\langle A_{11}\left(\rho_{f},\rho_{c}\right)\partial_{\alpha \alpha }g^{A}\rangle {\ddot{Q}}^{A}+\langle A_{12}\left(\rho_{f},\rho_{c}\right)\rangle \partial_{\alpha }{\ddot{\Theta }}_{\alpha }+\\ +\langle A_{12}\left(\rho_{f},\rho_{c}\right)\partial_{\alpha }h_{\alpha }^{B}\rangle {\ddot{\Phi }}_{\alpha }^{B}+\langle B_{1}\rangle \ddot{W} & =\langle q/h_{c}^{3}\rangle ,\\ \langle C_{\alpha \beta \gamma \delta }\rangle \partial_{\alpha \beta \gamma }W+\langle C_{\alpha \beta \gamma \delta }\partial_{\alpha \beta \gamma }g^{A}\rangle Q^{A}-\langle {\hat{C}}_{\alpha \beta \gamma \delta }\rangle \partial_{\alpha \gamma }\Theta_{\beta }-\langle {\hat{C}}_{\alpha \beta \gamma \delta }\partial_{\alpha \gamma }h_{\beta }^{B}\rangle \Phi_{\beta }^{B}+\\ -\langle A_{11}\left(\rho_{f},\rho_{c}\right)\rangle \partial_{\delta }\ddot{W}-\langle A_{11}\left(\rho_{f},\rho_{c}\right)\partial_{\delta }g^{A}\rangle {\ddot{Q}}^{A}+\langle A_{12}\left(\rho_{f},\rho_{c}\right)\rangle {\ddot{\Theta }}_{\delta }+\\ +\langle A_{12}\left(\rho_{f},\rho_{c}\right)h_{\delta }^{B}\rangle {\ddot{\Phi }}_{\delta }^{B}+\langle B_{2}\rangle \Theta_{\delta } & =\langle m_{\delta }/h_{c}^{3}\rangle ,\\ \langle C_{\alpha \beta \gamma \delta }g^{K}\rangle \partial_{\alpha \beta \gamma \delta }W+\langle C_{\alpha \beta \gamma \delta }\partial_{\alpha \beta \gamma \delta }g^{A}g^{K}\rangle Q^{A}-\langle {\hat{C}}_{\alpha \beta \gamma \delta }g^{K}\rangle \partial_{\alpha \beta \gamma }\Theta_{\delta }+\\ -\langle {\hat{C}}_{\alpha \beta \gamma \delta }\partial_{\alpha \beta \gamma }h_{\delta }^{B}g^{K}\rangle \Phi_{\delta }^{B}-\langle A_{11}\left(\rho_{f},\rho_{c}\right)g^{K}\rangle \partial_{\alpha \alpha }\ddot{W}-\langle A_{11}\left(\rho_{f},\rho_{c}\right)\partial_{\alpha \alpha }g^{A}g^{K}\rangle {\ddot{Q}}^{A}+\\ +\langle A_{12}\left(\rho_{f},\rho_{c}\right)g^{K}\rangle \partial_{\alpha }{\ddot{\Theta }}_{\alpha }+\langle A_{12}\left(\rho_{f},\rho_{c}\right)\partial_{\alpha }h_{\alpha }^{B}g^{K}\rangle {\ddot{\Phi }}_{\alpha }^{B}+\langle B_{1}g^{A}g^{K}\rangle {\ddot{Q}}^{A} & =\langle qg^{K}/h_{c}^{3}\rangle ,\\ \langle C_{\alpha \beta \gamma \delta }h_{\delta }^{L}\rangle \partial_{\alpha \beta \gamma }W+\langle C_{\alpha \beta \gamma \delta }\partial_{\alpha \beta \gamma }g^{A}h_{\delta }^{L}\rangle Q^{A}-\langle {\hat{C}}_{\alpha \beta \gamma \delta }h_{\delta }^{L}\rangle \partial_{\alpha \gamma }\Theta_{\beta }+\\ -\langle {\hat{C}}_{\alpha \beta \gamma \delta }\partial_{\alpha \gamma }h_{\beta }^{B}h_{\delta }^{L}\rangle \Phi_{\beta }^{B}-\langle A_{11}\left(\rho_{f},\rho_{c}\right)h_{\delta }^{L}\rangle \partial_{\delta }\ddot{W}-\langle A_{11}\left(\rho_{f},\rho_{c}\right)\partial_{\delta }g^{A}h_{\delta }^{L}\rangle {\ddot{Q}}^{A}+\\ +\langle A_{12}\left(\rho_{f},\rho_{c}\right)h_{\delta }^{L}\rangle {\ddot{\Theta }}_{\delta }+\langle A_{12}\left(\rho_{f},\rho_{c}\right)h_{\delta }^{B}h_{\delta }^{L}\rangle {\ddot{\Phi }}_{\delta }^{B}+\langle B_{2}h_{\delta }^{B}h_{\delta }^{L}\rangle \Phi_{\delta }^{B} & =\langle m_{\delta }h_{\delta }^{L}/h_{c}^{3}\rangle ,\\ \langle B_{1}g^{A}\rangle =0,\;\langle B_{2}h_{\alpha }^{B}\rangle =0,\;\alpha ,\beta ,\gamma ,\delta =1,2,\;A,K=1,2,\ldots ,N,\;B,L & =1,2,\ldots ,M, \end{array}$$

where in Equations (8)_2,4_ there is no summation over δ. The above system of equations is a system of 3+N+M equations with constant coefficients, which can be solved using well-known mathematic methods. The greatest advantage of the presented solution is that the same model can be theoretically used to analyse any type of microheterogeneous sandwich plate, which can be useful during the optimisation process.

## 6. Free Vibration Analysis of Sandwich Plate with Honeycomb Core

In this section, free vibration frequencies of a three-layered sandwich plate with honeycomb core are evaluated using the derived tolerance model (8) and validated with the use of FEM analysis.

**Figure 4:**
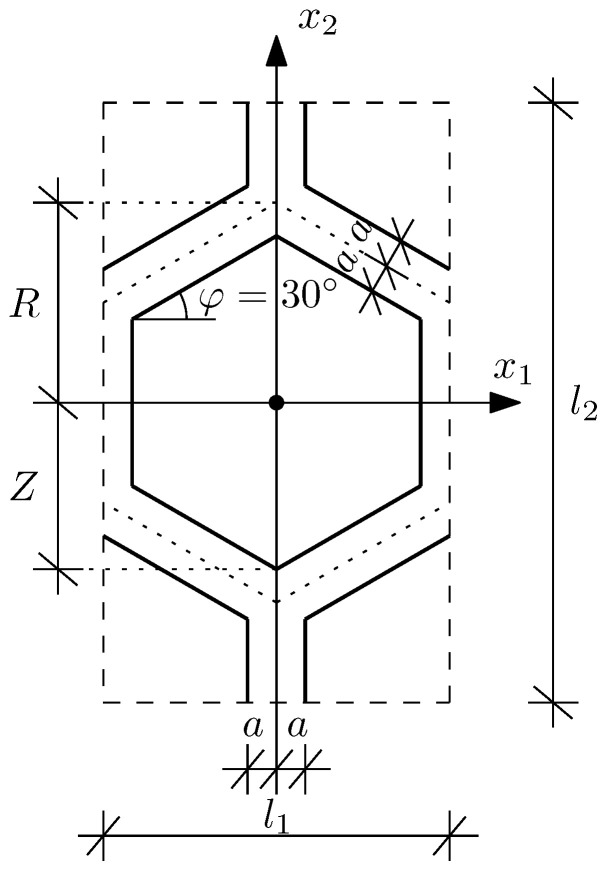
The details of a basic periodicity cell of the core.

The considered structure is a three-layered sandwich plate simply supported on all four edges. Let us assume that its characteristic dimensions along the $x_{1}$ and $x_{2}$ axis directions are equal to $L_{1}$ and $L_{2}$, respectively. The thickness of every layer is constant throughout the structure and equal to $h_{f}\left(x\right)=h_{f}$ and $h_{c}\left(x\right)=h_{c}$, hence $H\left(x\right)=2h_{f}+h_{c}=H$. Additionally, it is assumed that every layer is made of isotropic materials only. Additionally, it should be emphasised that the structure consists of homogeneous outer layers and a core with a periodic honeycomb microstructure, cf. Figure 1. The details of the basic periodicity cell defined by the core microstructure, together with a physical sense of the introduced denotations used to describe it, are presented in Figure 4.

**Figure 5:**
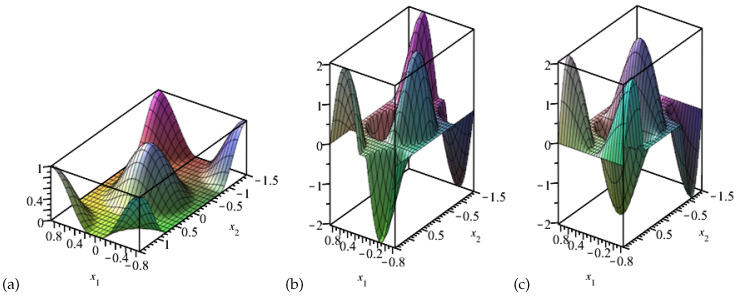
Visualisation of the assumed fluctuation shape functions: (**a**) ${\tilde{g}}^{1}$, (**b**) ${\tilde{h}}_{1}^{1}$, (**c**) ${\tilde{h}}_{2}^{1}$.

It can be easily noticed that the investigated structure fulfills all initial conditions of the derived averaged model. Hence, it can be used in its vibration analysis. In order to obtain free vibration frequencies of the described structure, all external loadings in (8) must be neglected. In the second step, a set of fluctuation shape functions must be assumed. The main aim of introducing such functions is the modelling of microscale disturbances in displacement fields caused by periodic microstructure, hence those functions should be properly adjusted. The most accurate way of obtaining them is solving the eigenvalue issue on the basic periodicity cell with, for example, the FEM analysis. However, the application of those exact functions, derived specially for a specific periodicity cell, is usually inconvenient in a large scale optimisation process. That it why, in this work, an approximation of those functions is introduced in the following form:

(9) $$\begin{array}{cc} {\tilde{g}}^{1}\left(x\right) & =\left\{\begin{array}{cc} \frac{1}{2}\left[\cos\left(\frac{\pi }{Z}\sqrt{x_{1}^{2}+x_{2}^{2}}\right)+1\right] & for\;x_{1}^{2}+x_{2}^{2}\leq Z^{2}\\ \frac{1}{2}\left[\cos\left(\frac{\pi }{Z}\sqrt{{\left(x_{1}-\frac{\sqrt{3}}{2}R\right)}^{2}+\left(x_{2}-\frac{3}{2}R\right)}\right)+1\right] & for\;{\left(x_{1}-\frac{\sqrt{3}}{2}R\right)}^{2}+{\left(x_{2}-\frac{3}{2}R\right)}^{2}\leq Z^{2}\\ \frac{1}{2}\left[\cos\left(\frac{\pi }{Z}\sqrt{{\left(x_{1}-\frac{\sqrt{3}}{2}R\right)}^{2}+\left(x_{2}+\frac{3}{2}R\right)}\right)+1\right] & for\;{\left(x_{1}-\frac{\sqrt{3}}{2}R\right)}^{2}+{\left(x_{2}+\frac{3}{2}R\right)}^{2}\leq Z^{2}\\ \frac{1}{2}\left[\cos\left(\frac{\pi }{Z}\sqrt{{\left(x_{1}+\frac{\sqrt{3}}{2}R\right)}^{2}+\left(x_{2}-\frac{3}{2}R\right)}\right)+1\right] & for\;{\left(x_{1}+\frac{\sqrt{3}}{2}R\right)}^{2}+{\left(x_{2}-\frac{3}{2}R\right)}^{2}\leq Z^{2}\\ \frac{1}{2}\left[\cos\left(\frac{\pi }{Z}\sqrt{{\left(x_{1}+\frac{\sqrt{3}}{2}R\right)}^{2}+\left(x_{2}+\frac{3}{2}R\right)}\right)+1\right] & for\;{\left(x_{1}+\frac{\sqrt{3}}{2}R\right)}^{2}+{\left(x_{2}+\frac{3}{2}R\right)}^{2}\leq Z^{2}\\ 0 & otherwise \end{array}\right.\\ {\tilde{h}}_{1}^{1}\left(x\right) & =\partial_{1}{\tilde{g}}^{1}\left(x\right),\;\;{\tilde{h}}_{2}^{1}\left(x\right)=\partial_{2}{\tilde{g}}^{1}\left(x\right), \end{array}$$

hence, A,K=1 and B,L=1. The visualisation of the proposed fluctuation shape functions is presented in Figure 5. It can be noticed that the normalising conditions $\langle B_{1}g^{A}\rangle =0,\langle B_{2}h_{\alpha }^{B}\rangle =0$ are instantly satisfied by those functions.

Apart from fluctuation shape functions, one should also assume the form of a solution of unknown displacement fields. In case of plate, which is simply supported on all four edges, it is possible to predict this form in a way that satisfies all the boundary conditions:

(10) $$\begin{array}{cc} W(x,t) & =A_{W}\sin\left(n\pi x_{1}/L_{1}\right)\sin\left(m\pi x_{2}/L_{2}\right)\sin\left(\omega t\right),\\ \Theta_{1}\left(x,t\right) & =A_{\Theta_{1}}\cos\left(n\pi x_{1}/L_{1}\right)\sin\left(m\pi x_{2}/L_{2}\right)\sin\left(\omega t\right),\\ \Theta_{2}\left(x,t\right) & =A_{\Theta_{2}}\sin\left(n\pi x_{1}/L_{1}\right)\cos\left(m\pi x_{2}/L_{2}\right)\sin\left(\omega t\right),\\ Q^{1}\left(x,t\right) & =A_{Q}\sin\left(n\pi x_{1}/L_{1}\right)\sin\left(m\pi x_{2}/L_{2}\right)\sin\left(\omega t\right),\\ \Phi_{\alpha }^{1}\left(x,t\right) & =A_{\Phi_{\alpha }^{1}}\sin\left(n\pi x_{1}/L_{1}\right)\sin\left(m\pi x_{2}/L_{2}\right)\sin\left(\omega t\right),\;\alpha =1,2, \end{array}$$

where $A_{W},A_{\Theta_{1}},A_{\Theta_{2}},A_{Q},A_{\Phi_{1}^{1}},A_{\Phi_{2}^{1}}$ are unknown amplitudes of vibrations, n,m are wave numbers and ω is a free vibration angular frequency. By introducing definitions (10) to the system of Equations (8), one obtains a simple set of six algebraic equations, which properly transformed yield-free vibration frequencies of the considered structure. Due to recent reports, that taking into considerations fluctuations of both in-plane and vertical displacements may lead to imprecise results, cf. [38], in our investigations, two different cases of tolerance model are considered. In Case I it is assumed that in-plane fluctuations have negligibly small amplitudes, hence, they can be neglected:

$$g^{1}\left(x\right)\equiv {\tilde{g}}^{1}\left(x\right),\;h_{1}^{1}\equiv 0,\;h_{2}^{1}\equiv 0,$$

while in Case II the vertical fluctuations are negligibly small when compared to in-plane fluctuations:

$$g^{1}\left(x\right)\equiv 0,\;h_{1}^{1}\left(x\right)\equiv {\tilde{h}}_{1}^{1}\left(x\right),\;h_{2}^{1}\left(x\right)\equiv {\tilde{h}}_{2}^{1}\left(x\right).$$

Consequently, the algebraic system of equations in Case I consists of four equations only (equations derived basing on Formula (8)_4_ are equivalent to zero), while Case II consists of five equations (equation derived basing on Formula (8)_3_ is equivalent to zero).

The tolerance model of vibrations of the three-layered sandwich plate, which takes into considerations all of the above assumptions, is used to evaluate free vibrations of 36 different sandwich structures. Each of them is also investigated using an FEM model created in Abaqus. Within this method the whole structure is modelled with the use of eight-node brick elements with reduced integration (C3D8R) and proper boundary conditions. The core of the structure is analysed using the sweep mesh, while the faces—the structured mesh. For both parts, the approximated global size of elements is assumed to be 0.02 m, which guarantees a good convergence of the results. In order to present the results in a concise form, only the relative errors between tolerance models and FEM models are shown in Table 1, Table 2, Table 3 and Table 4. The relative error between the Tolerance Model in Case I and FEM model is denoted as C1, while C2 stands for the relative error between the Tolerance Model in Case II and the FEM model. Common dimensions and material properties of the structures are listed below:

$$\begin{array}{ccc} E_{f}=210\;\mathrm{GPa} & \nu_{f}=0.3, & \rho_{f}=7850\;{\mathrm{kg}/m}^{3},\\ E_{c}=5\;\mathrm{GPa}, & \nu_{c}=0.3, & \rho_{c}=500\;{\mathrm{kg}/m}^{3},\\ h_{f}=0.0025\;m, & R=0.05\;m, & Z=R-\frac{2\sqrt{3}}{3}a,\\ l_{1}=\sqrt{3}R, & l_{2}=3R, \end{array}$$

while the rest of the characteristic dimensions, such as the thickness of the core $h_{c}$ and thickness of the vertical wall of hex *a*, are specified in Table 1, Table 2, Table 3 and Table 4. Eventually, all calculations are performed for three different dimensions of the structure, denoted as follows:

- Size I (S1)—$L_{1}=10l_{1},L_{2}=10l_{2};$
- Size II (S2)—$L_{1}=20l_{1},L_{2}=20l_{2};$
- Size III (S3)—$L_{1}=40l_{1},L_{2}=20l_{2};$

and within several different modes, distinguished by wave numbers:

- Mode I—n=1,m=1;
- Mode II—n=1,m=2;
- Mode III—n=1,m=3;
- Mode IV—n=2,m=1;
- Mode V—n=2,m=2;
- Mode VI—n=3,m=3.

The results presented in Table 1, Table 2, Table 3 and Table 4 should be properly interpreted, so several hints for modelling the vibrations of sandwich structures can be made. Let us formulate them in points.

- The relative errors between the results tend to decrease as the parameter *a* (representing the thickness of the walls of honeycomb in the periodicity cell) raises. It should be noticed that the initially assumed in-plane displacement field, cf. (1), is dedicated to sandwich structures with cores filling the whole available space between faces. In the case of ’thick’ honeycomb core, characterised by high values of parameter *a*, this assumption is still applicable, which results in generally satisfactory convergence of results. In the case of ’thin’ honeycomb, this assumption is slowly corrupting. As a consequence, the relative errors between averaged solution and FEM can reach up to 25% (cf. a=0.005m,S1).
- There are significant differences in the results of the averaged models in Case I and Case II. The only difference between those cases is a set of fluctuation shape functions. It can be noticed that for structures with lower thickness of the core $h_{c}$, the assumption of negligibly small fluctuations of vertical displacements (Case II) is applicable. Hence, it produces results, which in general are convergent with the FEM analysis. Meanwhile, for higher values of the thickness $h_{c}$, it seems that the fluctuations of vertical displacements are more influential, hence they cannot be neglected (Case I).
- In order to obtain precise results within the averaged model, the considered structure should be made of a sufficiently large quantity of periodicity cells. It can be noticed that for relatively small periodic structures (S1), the convergence of results in Case II is usually worse than in the case of larger structures (S2,S3). It can be caused by boundary conditions, which produce disturbances in displacement fields on a considerable span of the plate. A similar remark can be made for Case I, excluding structures with a low thickness of the core $h_{c}$, for which such a set of fluctuation shape functions is not applicable.
- Another reason for lower accuracy of results in the case of small periodic structures (S1) can be the issue of slowly varying functions. Based on the definitions, a slowly varying function is a function, which is ’almost’ constant on any periodicity cell, with respect to a certain tolerance parameter δ. In the case of small periodic structures, this assumption can be satisfied only for higher values of parameter δ, which results in lower accuracy of the proposed solution.
- It can be noticed that in several cases the discrepancies in the results between the averaged model and FEM analysis tend to raise higher modes of vibrations. The reason for this phenomenon can be also connected with difficulties in satisfying the condition of nearly constant values of slowly varying functions on a basic periodicity cell. In the case of higher modes of vibrations, this condition must yield higher values of tolerance parameter δ and, consequently, lower accuracy of the obtained results. Nevertheless, with a properly adjusted fluctuation shape functions, the averaged model can be used to estimate several basic free vibration frequencies of any of the analysed sandwich structures.

## 7. Final Remarks

In this article, the application of the tolerance averaging technique in the vibration analysis of a three-layered sandwich plate with a honeycomb core is presented. Despite the periodic microstructure of the core, the derived averaged model of the considered plate, based on the zig-zag hypothesis, is characterised by constant coefficients. Since the analytical solution to such a system of governing equations is relatively simple to obtain, this feature should be considered as the greatest finding of this work. Moreover, the proposed solution takes into consideration the microscale fluctuations of displacements. This feature is unreachable for other analytical methods, such as an asymptotic homogenisation method or other methods based on evaluation of effective properties of the heterogeneous layers.

In the calculation example, the free vibration analysis of the three-layered structure with basic honeycomb core is performed. Based on the results shown in Table 1, Table 2, Table 3 and Table 4, one can conclude that the presented averaged solution is capable of providing reasonably precise results. However, their convergence with FEM analysis is highly dependent on several factors, such as the introduced fluctuation shape functions or the dimensions of the structure. As a consequence, the application of the averaged models in the analysis of such structures requires either ’an engineering intuition’ or a previous experience in tolerance modelling, which stands for an unquestionable drawback of the presented solution.

On the other hand, one can notice that there is a significant difference in computing time of the proposed analytical solution and FEM models. In the case of the tolerance model, most time-consuming calculations are connected with the evaluation of coefficients of the governing Equations (8). Depending on the amount of the assumed fluctuation shape functions those calculations can require more or less computing power. However, it can be noticed that afterward the obtained coefficients can be used to analyse multiple structures with different characteristic dimensions, as long as the shape of the inhomogeneity remains constant. Those multiple investigations are usually instantly performed even by basic modern computers. Meanwhile, the creation of the geometry of the microperiodic FEM model is already a time-consuming process. Moreover, due to the assumed inhomogeneities, those models usually require a highly refined mesh, in order to provide reliable results. As a consequence, the computing resources required to perform the analysis of a single structure within FEM is incomparably higher, than in the case of the presented averaged model. Hence, it can be stated that the derived analytical solution to the issue of vibrations of the microheterogeneous sandwich plate is an efficient, time-saving option of the analysis, when compared to the FEM numerical calculations.

Moreover, one should notice that the analytical solution brings many opportunities, which are unreachable for numerical models. During the evaluation of coefficients of the governing equations, one can assume certain properties of the structure as parameters. As a consequence, it is possible to derive a calculation algorithm, which instantly produce relations between certain results and those parameters, in a large scope of calculation cases. Such relations are extremely useful during the optimisation process.

Moreover, let us emphasise that the presented solution is suitable for any kind of periodic microstructure of the core or faces. Hence, any type of periodic sandwich plate can be modelled with exactly the same calculation algorithm. In the case of the sandwich plates with honeycomb core, it is particularly important as honeycombs can differ from each other not only with dimensions *a*, *R* and *Z*, but also with an angle φ (cf. Figure 4) or even they can have different thicknesses of vertical and skew walls. In the case of tolerance modelling, all those geometries require the proper adjustment of the periodicity cell’s definition and fluctuation shape functions only. Hence, the presented solution can be considered a convenient tool for the optimisation process. In very special cases, for example, auxetic honeycombs, some additional adjustments may be required, but such structures were not yet investigated.

Eventually, let us state that in order to validate the proposed averaged solution a comparison with experimental results should be made. Such work will be carried out in the nearest future.

## Figures and Tables

**Table 1:** The relative errors between averaged models and FEM model for a=0.005m.

$h_{c}\left[\mathrm{mm}\right]$	Mode	S1		S2		S3	
		C1	C2	C1	C2	C1	C2
100	I	10.7%	16.9%	2.0%	8.8%	−1.7%	5.4%
	II	14.1%	20.0%	5.6%	12.1%	3.5%	10.2%
	III	16.9%	22.7%	9.3%	15.6%	8.3%	14.7%
	IV	16.1%	22.0%	9.1%	15.4%	2.0%	8.8%
	V	16.8%	22.6%	10.5%	16.7%	5.7%	12.2%
	VI	18.8%	24.5%	14.6%	20.5%	10.7%	16.9%
50	I	4.6%	11.8%	−3.1%	4.7%	−5.6%	2.4%
	II	8.1%	15.1%	−0.5%	7.2%	−1.9%	5.8%
	III	11.7%	18.4%	2.9%	10.3%	2.2%	9.6%
	IV	11.5%	18.2%	3.2%	10.6%	−3.1%	4.8%
	V	11.9%	18.6%	4.4%	11.7%	−0.4%	7.3%
	VI	14.1%	20.7%	9.2%	16.1%	4.4%	11.7%
25	I	−1.5%	6.6%	−6.8%	1.7%	−8.4%	0.2%
	II	1.6%	9.4%	−5.4%	3.0%	−5.6%	2.8%
	III	5.6%	13.1%	−2.6%	5.6%	−2.4%	5.8%
	IV	5.4%	12.9%	−1.9%	6.2%	−6.7%	1.7%
	V	5.2%	12.7%	−1.6%	6.5%	−5.2%	3.1%
	VI	7.3%	14.7%	2.6%	10.4%	−1.8%	6.3%

**Table 2:** The relative errors between averaged models and FEM model for a=0.010m.

$h_{c}\left[\mathrm{mm}\right]$	Mode	S1		S2		S3	
		C1	C2	C1	C2	C1	C2
100	I	4.7%	10.2%	0.2%	6.0%	−1.3%	4.5%
	II	7.0%	12.3%	1.9%	7.6%	0.9%	6.7%
	III	9.0%	14.3%	3.9%	9.4%	4.4%	9.9%
	IV	9.5%	14.7%	4.1%	9.7%	0.1%	5.9%
	V	10.0%	15.3%	4.8%	10.3%	1.8%	7.5%
	VI	12.6%	17.8%	8.0%	13.4%	4.9%	10.4%
50	I	−0.3%	6.0%	−3.7%	2.8%	−4.7%	1.8%
	II	1.7%	7.8%	−2.6%	3.8%	−3.1%	3.3%
	III	4.0%	10.0%	−1.1%	5.2%	−1.0%	5.3%
	IV	4.9%	10.9%	−0.6%	5.7%	−3.7%	2.7%
	V	5.3%	11.2%	−0.2%	6.0%	−2.7%	3.7%
	VI	8.2%	14.0%	3.0%	9.1%	−0.2%	6.1%
25	I	−4.6%	2.1%	−6.4%	0.5%	−7.1%	−0.2%
	II	−3.7%	3.0%	−6.1%	0.7%	−5.8%	1.1%
	III	−1.7%	4.9%	−5.0%	1.8%	−4.3%	2.4%
	IV	−0.2%	6.2%	−4.2%	2.6%	−6.4%	0.5%
	V	−0.7%	5.8%	−4.6%	2.2%	−6.2%	0.7%
	VI	1.7%	8.1%	−2.5%	4.1%	−4.8%	2.0%

**Table 3:** The relative errors between averaged models and FEM model for a=0.015m.

$h_{c}\left[\mathrm{mm}\right]$	Mode	S1		S2		S3	
		C1	C2	C1	C2	C1	C2
100	I	1.4%	6.7%	−1.4%	4.0%	−2.1%	3.4%
	II	3.2%	8.3%	−0.4%	5.0%	−0.9%	4.5%
	III	4.9%	10.0%	0.9%	6.2%	0.6%	5.9%
	IV	4.6%	9.7%	0.7%	6.0%	−1.4%	4.0%
	V	5.2%	10.3%	1.3%	6.5%	−0.4%	5.0%
	VI	7.1%	12.1%	3.5%	8.6%	1.5%	6.7%
50	I	−2.5%	3.3%	−4.1%	1.8%	−4.5%	1.4%
	II	−1.2%	4.5%	−3.7%	2.2%	−3.8%	2.1%
	III	0.4%	6.1%	−2.8%	3.0%	−2.8%	3.0%
	IV	0.0%	5.7%	−2.9%	2.9%	−4.2%	1.7%
	V	0.5%	6.1%	−2.7%	3.1%	−3.7%	2.2%
	VI	2.0%	7.5%	−1.1%	4.6%	−2.5%	3.2%
25	I	−5.6%	0.5%	−6.2%	0.0%	−6.6%	−0.4%
	II	−5.2%	0.9%	−6.2%	−0.1%	−5.7%	0.5%
	III	−3.9%	2.1%	−5.5%	0.6%	−4.9%	1.2%
	IV	−4.0%	2.0%	−5.2%	0.9%	−6.2%	0.0%
	V	−4.4%	1.7%	−5.7%	0.5%	−6.3%	−0.1%
	VI	−4.1%	1.9%	−5.1%	1.0%	−5.8%	0.3%

**Table 4:** The relative errors between averaged models and FEM model for a=0.020m.

$h_{c}\left[\mathrm{mm}\right]$	Mode	S1		S2		S3	
		C1	C2	C1	C2	C1	C2
100	I	1.2%	3.7%	−0.5%	2.0%	−1.1%	1.4%
	II	2.1%	4.5%	-0.2%	2.4%	−0.6%	1.9%
	III	3.0%	5.5%	0.5%	3.0%	0.2%	2.7%
	IV	4.0%	6.4%	1.1%	3.5%	−0.5%	2.0%
	V	4.2%	6.6%	1.3%	3.7%	−-0.2%	2.3%
	VI	6.1%	8.5%	2.9%	5.4%	1.0%	3.5%
50	I	−1.6%	1.3%	−2.4%	0.5%	−2.7%	0.3%
	II	−1.1%	1.8%	−2.3%	0.7%	−2.3%	0.7%
	III	−0.3%	2.6%	−1.9%	1.1%	−1.9%	1.1%
	IV	0.3%	3.1%	−1.5%	1.4%	−2.4%	0.5%
	V	0.3%	3.2%	−1.6%	1.4%	−2.3%	0.7%
	VI	1.7%	4.5%	−0.6%	2.3%	−1.7%	1.2%
25	I	−3.9%	−0.7%	−4.0%	−0.8%	−4.4%	−1.1%
	II	−4.1%	−0.9%	−4.3%	−1.0%	−2.7%	0.5%
	III	−3.5%	−0.3%	−3.9%	−0.7%	−3.2%	0.0%
	IV	−2.7%	0.5%	−3.2%	0.0%	−4.0%	−0.8%
	V	−3.5%	−0.3%	−3.8%	−0.6%	−4.3%	−1.1%
	VI	−3.2%	0.0%	−3.6%	−0.4%	−4.2%	−1.0%

## Data Availability

Not applicable.

## References

[1] Tewari K., Pandit M., Budarapu P., Natarajan S. (2022). Analysis of sandwich structures with corrugated and spiderweb-inspired cores for aerospace applications. Thin-Walled Struct..

[2] Kheyabani A., Massarwa E., Kefal A. (2022). Multiscale structural analysis of thick sandwich structures using parametric HFGMC micromechanics and isogeometric plate formulation based on refined zigzag theory. Compos. Struct..

[3] Chen Y., Ma H., Li A., Fang H., Liu Y., Li H. (2022). Hydroelastic analysis of double-segment floating sandwich structures under wave action. Ocean Eng..

[4] Deng Y., Zhou N., Li X., Wang X., Wei G., Jia H. (2022). Dynamic response and failure mechanism of S-shaped CFRP foldcore sandwich structure under low-velocity impact. Thin-Walled Struct..

[5] Lee H.M., Kim D.H., Kim D.Y., Kim M.S., Park J., Yoon G.H. (2022). Enhancement of vibration attenuation and shock absorption in composite sandwich structures with porous foams and surface patterns. Compos. Struct..

[6] Pang Y., Yan X., Qu J., Wu L. (2022). Dynamic response of polyurethane foam and fiber orthogonal corrugated sandwich structure subjected to low-velocity impact. Compos. Struct..

[7] Alanbay B., Batra R. (2022). Optimization of blast mitigating sandwich structures with fiber-reinforced face sheets and PVC foam layers as core. Thin-Walled Struct..

[8] Hedayati R., Yousefi A., Bodaghi M. (2022). Sandwich structures with repairable cores based on truncated cube cells. Compos. Part B Eng..

[9] Hu H., Belouettar S., Potier-Ferry M., Daya E.M. (2008). Review and assessment of various theories for modeling sandwich composites. Compos. Struct..

[10] Hu H., Belouettar S., Potier-Ferry M., Makradi A., Koutsawa Y. (2011). Assessment of various kinematic models for instability analysis of sandwich beams. Eng. Struct..

[11] Carrera E., Brischetto S. (2009). A survey with numerical assessment of classical and refined theories for the analysis of sandwich plates. Appl. Mech. Rev..

[12] Iurlaro L., Gherlone M., Di Sciuva M., Tessler A. (2013). Assessment of the Refined Zigzag Theory for bending, vibration, and buckling of sandwich plates: A comparative study of different theories. Compos. Struct..

[13] Al-Furjan M., Shan L., Shen X., Kolahchi R., Rajak D.K. (2022). Combination of FEM-DQM for nonlinear mechanics of porous GPL-reinforced sandwich nanoplates based on various theories. Thin-Walled Struct..

[14] Magnucki K., Lewinski J., Far M., Michalak P. (2020). Three-point bending of an expanded-tapered sandwich beam - Analytical and numerical FEM study. Mech. Res. Commun..

[15] Lu Q., Liu C., Wang P. (2022). Band gap enhancement and vibration reduction of functionally graded sandwich metastructure beam. Compos. Struct..

[16] Xue B., Peng Y.X., Ren S.F., Liu N.N., Zhang Q. (2021). Investigation of impact resistance performance of pyramid lattice sandwich structure based on SPH-FEM. Compos. Struct..

[17] Park H. (2019). Investigation on low velocity impact behavior of sandwich composite and monolithic laminate plates using FEM analysis. Compos. Struct..

[18] Ge L., Zheng H., Li H., Liu B., Su H., Fang D. (2021). Compression behavior of a novel sandwich structure with bi-directional corrugated core. Thin-Walled Struct..

[19] Meng L., Lan X., Zhao J., Li H., Wang Z., Gao L. (2021). Failure analysis of bio-inspired corrugated sandwich structures fabricated by laser powder bed fusion under three-point bending. Compos. Struct..

[20] Li C.H., Yan J.B., Guan H.N. (2021). Finite element analysis on enhanced C-channel connectors in SCS sandwich composite structures. Structures.

[21] Ye X., Zhao C., He K., Zhou L., Li X., Wang J. (2021). Blast behaviors of precast concrete sandwich EPS panels: FEM and theoretical analysis. Eng. Struct..

[22] Gay D., Hoa S.V. (2007). Composite Materials. Design and Applications.

[23] Allen H.G. (1969). Analysis and Design of Structural Sandwich Panels.

[24] Malek S., Gibson L. (2015). Effective elastic properties of periodic hexagonal honeycombs. Mech. Mater..

[25] Woźniak C. (2010). Mathematical Modelling and Analysis in Continuum Mechanics of Microstructured Media.

[26] Jędrysiak J., Kaźmierczak-Sobińska M. (2020). Theoretical Analysis of Buckling for Functionally Graded Thin Plates with Microstructure Resting on an Elastic Foundation. Materials.

[27] Marczak J., Jędrysiak J. (2021). The Stability Analysis of Periodic Beams Interacting with Periodic Elastic Foundation with the Use of the Tolerance Averaging Technique. Materials.

[28] Jędrysiak J. (2020). Tolerance Modelling of Vibrations and Stability for Periodic Slender Visco-Elastic Beams on a Foundation with Damping. Revisiting. Materials.

[29] Tomczyk B., Bagdasaryan V., Gołąbczak M., Litawska A. (2021). On the modelling of stability problems for thin cylindrical shells with two-directional micro-periodic structure. Compos. Struct..

[30] Jędrysiak J. (2021). Non-asymptotic modelling of dynamics and stability for visco-elastic periodic beams on a periodic damping foundation. Compos. Struct..

[31] Domagalski Ł., Świątek M., Jędrysiak J. (2019). An analytical-numerical approach to vibration analysis of periodic Timoshenko beams. Compos. Struct..

[32] Domagalski Ł. (2018). Free and forced large amplitude vibrations of periodically inhomogeneous slender beams. Arch. Civ. Mech. Eng..

[33] Domagalski Ł. (2021). Comparison of the Natural Vibration Frequencies of Timoshenko and Bernoulli Periodic Beams. Materials.

[34] Ostrowski P., Jędrysiak J. (2021). Dependence of temperature fluctuations on randomized material properties in two-component periodic laminate. Compos. Struct..

[35] Wyczółkowski R., Bagdasaryan V., Tomczyk B. (2022). Modelling of effective thermal conductivity of a packed bed of steel bars with the use of chosen literature models. Compos. Struct..

[36] Kubacka E., Ostrowski P. (2021). A Finite Difference Algorithm Applied to the Averaged Equations of the Heat Conduction Issue in Biperiodic Composites—Robin Boundary Conditions. Materials.

[37] Kubacka E., Ostrowski P. (2021). Heat conduction issue in biperiodic composite using Finite Difference Method. Compos. Struct..

[38] Marczak J. (2020). On the correctness of results of averaged models of periodic sandwich plates depending on the set of fluctuation shape functions. Compos. Struct..

